# Automated assessment of periapical health based on the radiographic periapical index using YOLOv8, YOLOv11, and YOLOv12 one-stage object detection algorithms

**DOI:** 10.1038/s41598-025-21761-5

**Published:** 2025-10-20

**Authors:** Shehabeldin Saber, Hend Abou El Nasr, Ahmed A. Torky, Nora Saif

**Affiliations:** 1 Department of Endodontics, Faculty of Dentistry, The British University in Egypt, El Sherouk City, Egypt; 2 Dental Science Research Group, Health Research Centre of Excellence, The British University in Egypt, El Sherouk City, Egypt; 3 Department of Endodontics, Faculty of Dentistry, Cairo University, Cairo, Egypt; 4 Civil Engineering Department, Faculty of Engineering, The British University in Egypt, El-Sherouk City, Egypt; 5 Department of Oral and Maxillofacial Radiology, Faculty of Dentistry, Cairo University, Cairo, Egypt; 6 Egyptian Maxillofacial Radiology Alliance Lab – EMRA Lab, Cairo, Egypt

**Keywords:** Apical periodontitis, Periapical index, Artificial intelligence, Deep learning, Computer vision, YOLO, Dental radiology, Dental treatments, Machine learning

## Abstract

This study investigates the application of recent YOLO (You Only Look Once) algorithms for automated detection and classification of apical periodontitis using the Periapical Index (PAI) scoring system (1–5). A dataset of 699 digital periapical radiographs was collected from diverse sources, de-identified, and annotated by calibrated experts before splitting into training, validation, and testing sets. Three deep learning models (YOLOv8m, YOLOv11m, YOLOv12m) were trained and their performance was evaluated using Precision, Recall, F1 score, mean average precision (mAP50), Intersection over union (IoU), and confusion matrices. Two-sided McNemar’s exact test was further conducted to compare models on lesion-level outcomes. The results showed comparable mAP50 scores among the tested models. YOLOv11m and YOLOv12m demonstrated higher Precision (88.5% and 89.1%, respectively) compared to YOLOv8m (86.8%). YOLOv11m exhibited the highest Recall (86.2%) and maximum F1 score (87.1%). Confusion matrix analysis indicated superior prediction for PAI scores 3–5 across all models, with YOLOv11m excelling in scores 1 and 2, YOLOv8m performing best for score 4, and equal performance for score 5. The findings demonstrate the prospective of YOLO algorithms in automating the detection and classification of apical periodontitis using PAI. Accuracy and efficiency of tested models suggest their potential for integration in clinical workflows.

**Supplementary Information:**

The online version contains supplementary material available at 10.1038/s41598-025-21761-5.

## Introduction

Periapical health serves as a crucial indicator of pulp condition and a strong predictor of root canal treatment outcomes. Hence, periapical radiographs are fundamental for assessing the health of periapical tissues, forming the basis for endodontic diagnosis, treatment planning, and prognosis^1,2^. On dental radiographs, apical periodontitis can manifest in various forms, from a slight widening of the periodontal ligament space to a clearly visible bony lesion^3^.

This variability in radiographic presentation highlights the need for a standardized evaluation and reporting method to ensure accurate diagnosis and treatment planning in endodontic cases across different practitioners and settings. Various radiographic indices have been suggested for assessing the periapical status^4,5^, however, the periapical index (PAI) scoring system designed in 1986 by Ørstavik et al.^6^ is considered the most clinically reliable due its established histological correlation^7^. The PAI scoring system’s reliability and widespread adoption made it a valuable tool bridging the gap between research and clinical practice^5,8–10^. Still, radiographic interpretation remains subject to individual expertise^11^ and can be hindered by the superimposition of certain anatomical landmarks^12^. These limitations underscore the importance of ongoing research to develop automated assessment techniques to enhance diagnostic accuracy.

The use of convolutional neural networks (CNNs) in dental image analysis shows great potential for minimizing interpretative bias and improving diagnostic precision^13^. CNNs’ automated pattern recognition abilities are especially beneficial for identifying subtle abnormalities that may elude human observers^14^. CNNs have been successfully applied for classification, segmentation, and object detection across multiple conditions, including dental caries, periodontal bone loss, and oral cancer^15^. In the field of endodontics, CNNs have similarly demonstrated efficacy in tooth identification^15^, locating canals^16^, determination of apical terminus^17^, apical lesion detection^18^; as well as the detection of vertical root fracture^19^, and external cervical resorption^20^.

Extending beyond these applications, deep learning (DL) techniques have also shown promise in the diagnosis of periapical pathologies. Automated detection of apical periodontitis in 2D radiographs and cone beam computed tomography (CBCT) scans has been previously investigated in several studies using various algorithms^18–24^. A recent meta-analysis^2^ showed high accuracy of DL algorithms in detecting apical lesions, with a pooled sensitivity and specificity of 0.94 and 0.96, respectively. However, most studies have adopted a binary classification approach, focusing on assessing the presence or absence of apical periodontitis, rather than signifying disease progression or healing.

Advanced object detection algorithms, such as YOLO (You Only Look Once), renowned for their robustness and efficiency, present significant potential to further enhance capabilities and address complex challenges in dental imaging. Initially introduced in 2015, YOLO identifies objects by predicting bounding boxes and class probabilities directly from full images in a single evaluation^25^. Over the years, YOLO algorithms have evolved to process input images more effectively and improve feature integration across different scales. YOLOv8, developed by Ultralytics in 2023, incorporated several architectural improvements and optimizations, including newer and more efficient types of convolutional layers, to facilitate faster computation and better generalization^26^. More recently, YOLOv11 was introduced with modifications to increase spatial attention for accurately detecting small overlapping objects^27^. Finally, in 2025, architectural advancements were implemented in YOLOv12 to enhance training stability and model convergence^28^.

To our knowledge, limited research has explored the automated detection and classification of periapical lesions using primary architectures. Hirata et al.^29^ proposed a binary classification for PAI scores employing GoogLeNet, AlexNet, and ResNet CNNs. Moidu et al.^24^ employed the YOLOv3 model, which exhibited varying true prediction rates for different PAI scores, identifying challenges related to performance variability across different regions of the dental arch, which affected the model’s reliability in clinical applications. Conversely, Viet et al.^30^ focused on comparing the performance of YOLOv4 versus Faster R-CNN models, limiting their dataset to mandibular teeth only. Notably, all these studies sourced their data/radiographs from a single clinical facility and did not report external validation of the proposed models.

By leveraging architectural improvements and addressing the limitations of previous studies, the aim of our study was to develop a more robust and clinically applicable DL-based diagnostic tool to detect and classify AP using PAI in periapical radiographs, and to compare recent YOLO algorithms in terms of accuracy and speed using a diverse heterogeneous dataset from different clinical sources.

## Methods

All methods were carried out in accordance with relevant guidelines and regulations. All experimental protocols were approved by the research ethics committee of the Faculty of Dentistry at The British University in Egypt (Approval number: 24 − 004 on 10/01/2024). An informed consent for data usage was obtained from all subjects and/or their legal guardian(s).

### Data acquisition

A total of 699 digital periapical radiographs were retrospectively collected from the university clinic and two private clinics specialized in endodontics. The radiographs were acquired using two types of image plates; VistaScan (Dürr Dental, Bietigheim-Hissingen, Germany) (*n* = 249) and Soredex Digora Optime (Soredex, Tuusula, Finland) (*n* = 200), as well as an intra-oral sensor; EzSensor HD (Vatech, Hwaseong-si, Gyeonggi-do, Korea) (*n* = 250). These radiographs were acquired during routine diagnostic procedures or at any stage of endodontic treatment, without preference for specific teeth, age, gender, ethnicity, health condition, clinical symptoms, or tooth preference. Demographic analysis of the dataset disclosed a comparable gender distribution (56% females and 44% males), an age range of 12–78 years, and different ethnics.

### Sample size calculation

As the present study focused on developing a DL model, a priori sample size calculation was not applicable. Instead, all eligible periapical radiographs meeting the inclusion criteria were utilized to maximize model robustness. To estimate whether the sample size provided sufficient statistical sensitivity for detecting clinically meaningful differences, we performed a post-hoc power analysis using model classification accuracy as the primary outcome. The post-hoc analysis confirmed that model performance (F1 = 0.83–0.86) was significantly above chance (0.20) with > 99% power, indicating dataset adequacy.

### Data annotation

The collected digital radiographs were exported in JPEG format and de-identified manually, then scored according to the periapical index (PAI) described by Ørstavik et al.^6^. Scoring was performed by two endodontists (SS, HA) and one oral radiologist (NT) with more than 25 years of clinical and academic experience. The examiners were calibrated first by scoring 15% (*n* = 100) of the images. Images were viewed on an 18.5-inch HD LED monitor with a resolution of 1366 × 768. Zoom, brightness, and contrast tools were available for use. The examiners took a break of 5 min after scoring 10 radiographs. The radiographs were re-scored at a1-month interval to assess the intra- and inter-observer agreement, which was almost perfect (kappa values 0.97 and 0.96, respectively).

**Fig. 1:**
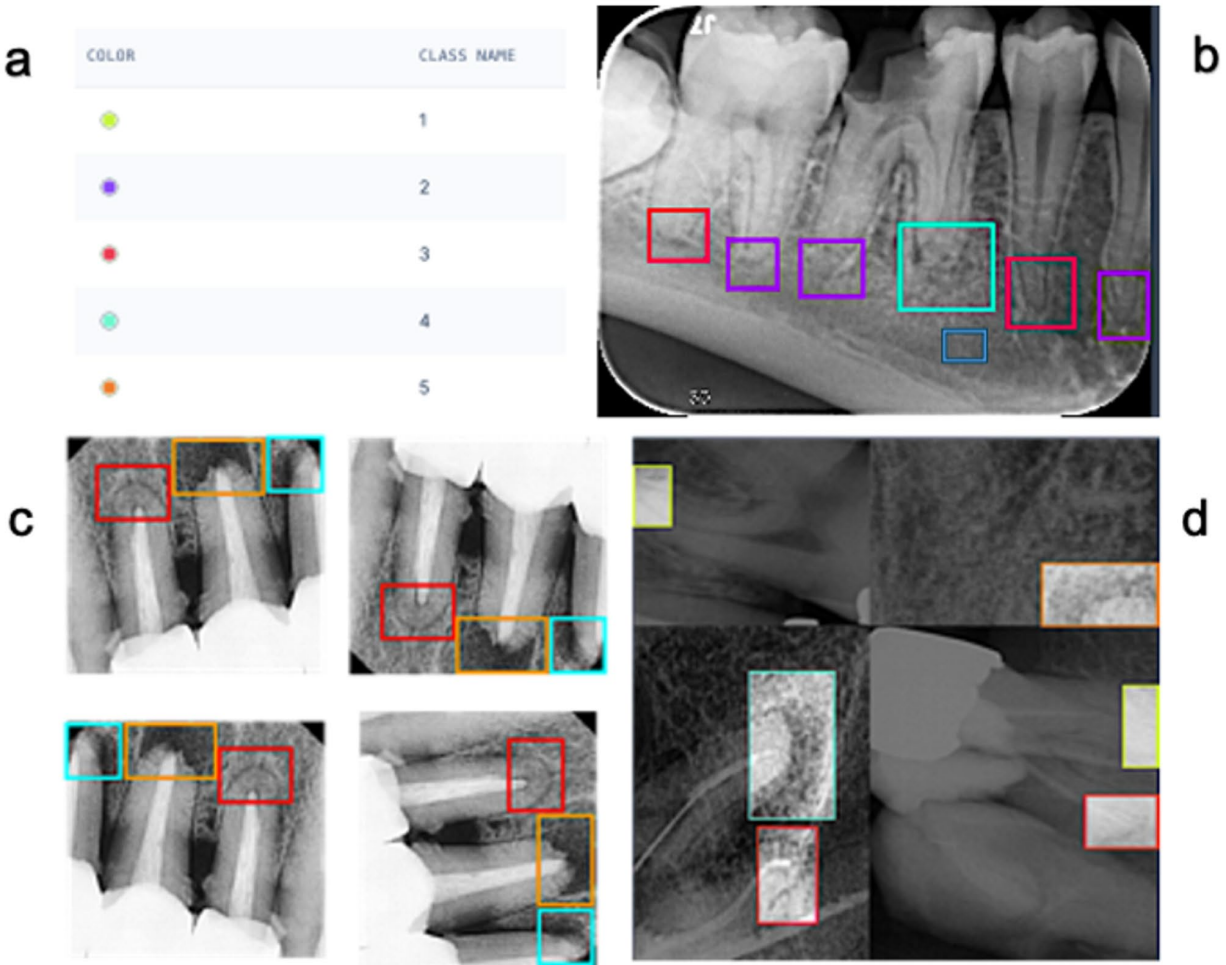
(**a**) assigned PAI scores, (**b**) manual annotation on Roboflow using the bounding box tool, (**c**) Geometric augmentation of the training dataset through altering pixel location, (**d**) Application of the mosaic pattern. Note that the increased brightness of boxes in (**d**) reflects the visualization overlay and is not an image-intensity augmentation.

For annotation, the dataset was uploaded to Roboflow^31^ which is an online platform for computer vision tasks. Two experts annotated the whole dataset independently, and the third was consulted in their presence to reach a consensus in case there was a scoring disagreement. In the annotation tool, experts manually drew colour-coded bounding boxes representative of the five PAI scores (Fig. 1a, b). In case of existing periapical lesions, the bounding boxes were drawn to include the lesion center, lesion border, associated root apex, and adjacent healthy bone.

### Data processing

The data were randomly split on image level into training (*n* = 475; 67.8%), validation (*n* = 139; 19.9%), and testing (*n* = 85; 12.2%) sets; the detailed distribution of PAI scores is presented in Supplementary Table 1. Before feeding the annotated radiographs into the DL model pipeline, they were digitally pre-processed. This included image cropping, auto-orientation, and resizing of median image size from (795 × 795) px to a generalized image size of (640 × 640) px. Cropping was performed automatically in Roboflow using the “crop to annotations” option. For each radiograph, the minimal rectangle enclosing the union of all ground-truth bounding boxes was retained—preserving all annotated regions while removing unannotated borders and corners. Additionally, all radiographs were resized using square letterbox to 640 × 640 (preserving the aspect ratio) and padded with a constant value (114); bounding boxes were re-mapped after scaling/padding.

Subsequently, image augmentation techniques were applied exclusively to the training set to improve dataset robustness. This included horizontal and vertical flipping, 90°, 180° and 270° rotation (Fig. 1c), as well as application of mosaic patterns (Fig. 1d). After augmentation, the training dataset became 4635 images with 23,413 annotations averaging 5.1 per image. The instances of PAI classes 1–5 were 6531, 4819, 4156, 4026, and 3881, respectively. Mosaic augmentation is considered as four-images in 2 × 2 collage with random scale/translation and labels transformed. The same pipeline was used for YOLOv8m/11m/12m.

### Model architecture

**Fig. 2:**
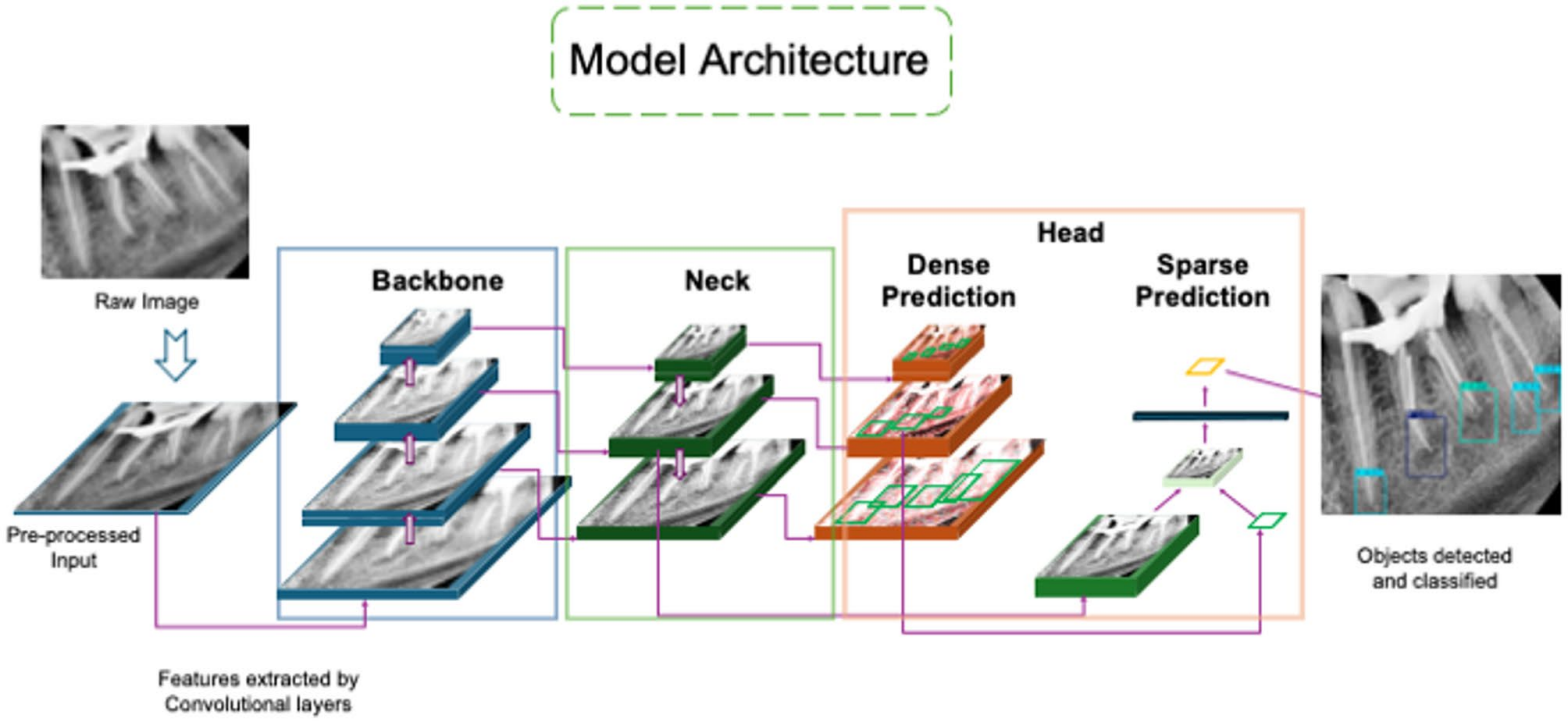
Simplified YOLO architecture of processing layers for object detection and classification.

The YOLO architecture has a modular structure, i.e., it is divided into three main components: backbone, neck, and head (Fig. 2). The backbone is responsible for extracting hierarchical features from input images, enabling the model to recognize patterns at various levels of detail. The neck refines these features through additional layers, enhancing edge detection and object localization. The head performs the detection task, where dense prediction layers use anchor boxes for comprehensive grid coverage, while sparse prediction layers focus on high-confidence regions to predict bounding boxes and class probabilities. This pipeline outputs radiographic images with annotated regions and classification labels. Given the dataset size, the medium (m) variant of each architecture—YOLOv8m, YOLOv11m, and YOLOv12 were selected, resulting in a total of three trained DL models.

### Model training, validation and testing

**Fig. 3:**
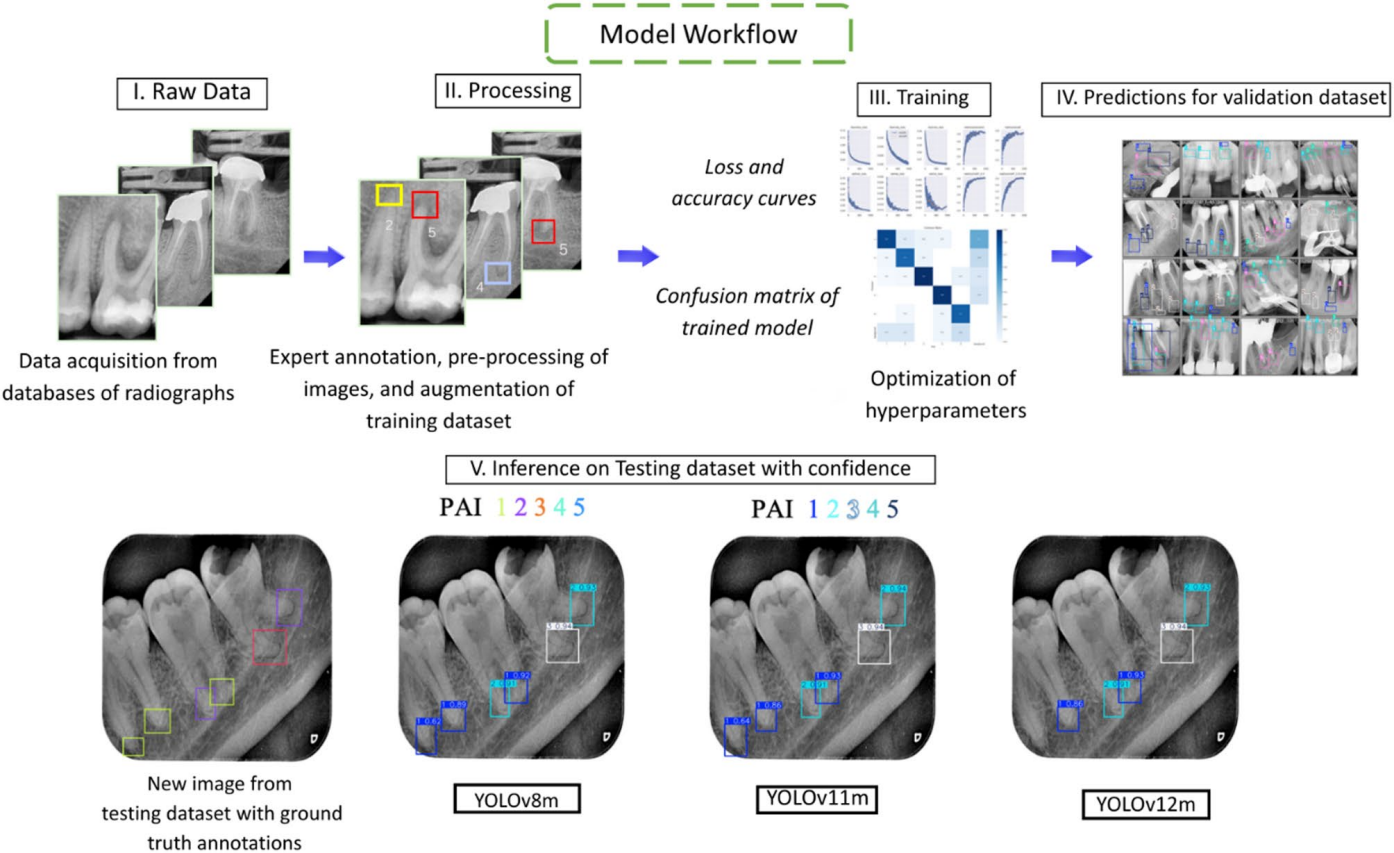
An illustration of the workflow used to train, validate, and test models.

The processed dataset was exported from Roboflow and fed into the end-to-end DL pipeline (Fig. 3). The models were trained for a maximum of 1000 epochs using the Adam optimizer with a learning rate of 0.001 and random weight initialization. A batch size of 50 images per epoch was employed. To prevent overfitting, early stopping was applied if model performance showed no improvement for 100 consecutive epochs. The optimized models were tested on data not seen by the model during training.

### External validation

The models were also externally validated against 70 new images acquired from a new source (private dental clinic) acquired using Planmeca Proscanner 2.0 (Planmeca Oy, Helsinki, Finland) and fulfilling the same inclusion criteria as the initial dataset to assess their generalizability. Demographic analysis of the external validation dataset also disclosed a comparable gender distribution (52% females and 48% males), an age range of 16–66 years, and different ethnics.

### Computational resources

Training, validating, and inference were conducted using the PyTorch library version 2.3.0 along with the default architectures of YOLOv8, YOLOv11, and YOLOv12 by Ultralytics. The computational infrastructure included two platforms—(1) Google Collaboratory platform using V100 NVIDIA GPU (12.7 GB system RAM, 15 GB GPU RAM, 78.2 GB disc space) with Python 3.8 on the Google Compute Engine backend, and (2) the Rescale cloud-computing platform, using a super-computing node with 2nd generation AMD EPYC processors running at 2.8 GHz and an NVIDIA A10 GPU.

### Evaluation metrics

The reporting of results and the selection of evaluation metrics were informed by the recently published “Core outcome measures in dental computer vision studies (DentalCOMS)’’^32^.

The following metrics were calculated:

- Precision (positive predictive value): the proportion of true positives to all predicted positives.
- Recall (sensitivity): the proportion of true positives to all actual positives.
- F1 score: a harmonic mean of precision and recall, computed as follows:
  $${\text{F1 Score}}\,=\,{\text{2}}\left( {{\text{Precision X Recall}}} \right)/({\text{Precision}}\,+\,{\text{Recall}})$$
- Mean average precision (*mAP50*): mean of average precision of each class, which is calculated as the area under the precision-recall curve at IoU ≥ 0.5.
- IoU (Intersection over Union): measures how much a predicted region overlaps the ground-truth region, calculated as the area of overlap divided by the area of union. It ranges from 0 to 1 (higher is better) and is typically thresholded (e.g., ≥ 0.5) to decide whether a detection counts as a match.

Furthermore, the confusion matrix across the 5 classes and the background were used in the evaluation of the testing dataset results. The detections were matched to the ground truth using a one-to-one, highest-IoU assignment with IoU ≥ 0.5 after non-maximum suppression (NMS) and a 0.25 confidence threshold. Unmatched predictions were counted as false positives (ground truth = background), and unmatched ground truth as false negatives (predicted = background).

## Results

The performance metrics of all trained models evaluated on the testing dataset, in addition to the number of convolutional layers, Trainable parameters, training epochs, total training time, Giga Floating Point Operations Per Second (GFLOPS), and processing time per image for all trained models are presented in Table 1.

**Table 1:** Results of accuracy metrics and the number of convolutional layers, trainable parameters, training epochs, total training time, giga floating point operations per second (GFLOPS), and processing time per image for all trained models.

	YOLOv8m	YOLOv11m	YOLOv12m
layers	92	125	169
Trainable parameters	25,842,655	20,033,887	20,108,767
Training epochs	421	521	399
Training time (hours)	12.140	9.870	10.350
GFLOPs	78.7	67.7	67.1
Processing time (ms)	3	5.4	6.8
mAP50	86.4%	86.6%	86.6%
Recall	83.5%	86.2%	84.4%
Precision	86.8%	88.5%	89.1%
Max F1 score (%)	85.2%	87.1%	86.6%

**Fig. 4:**
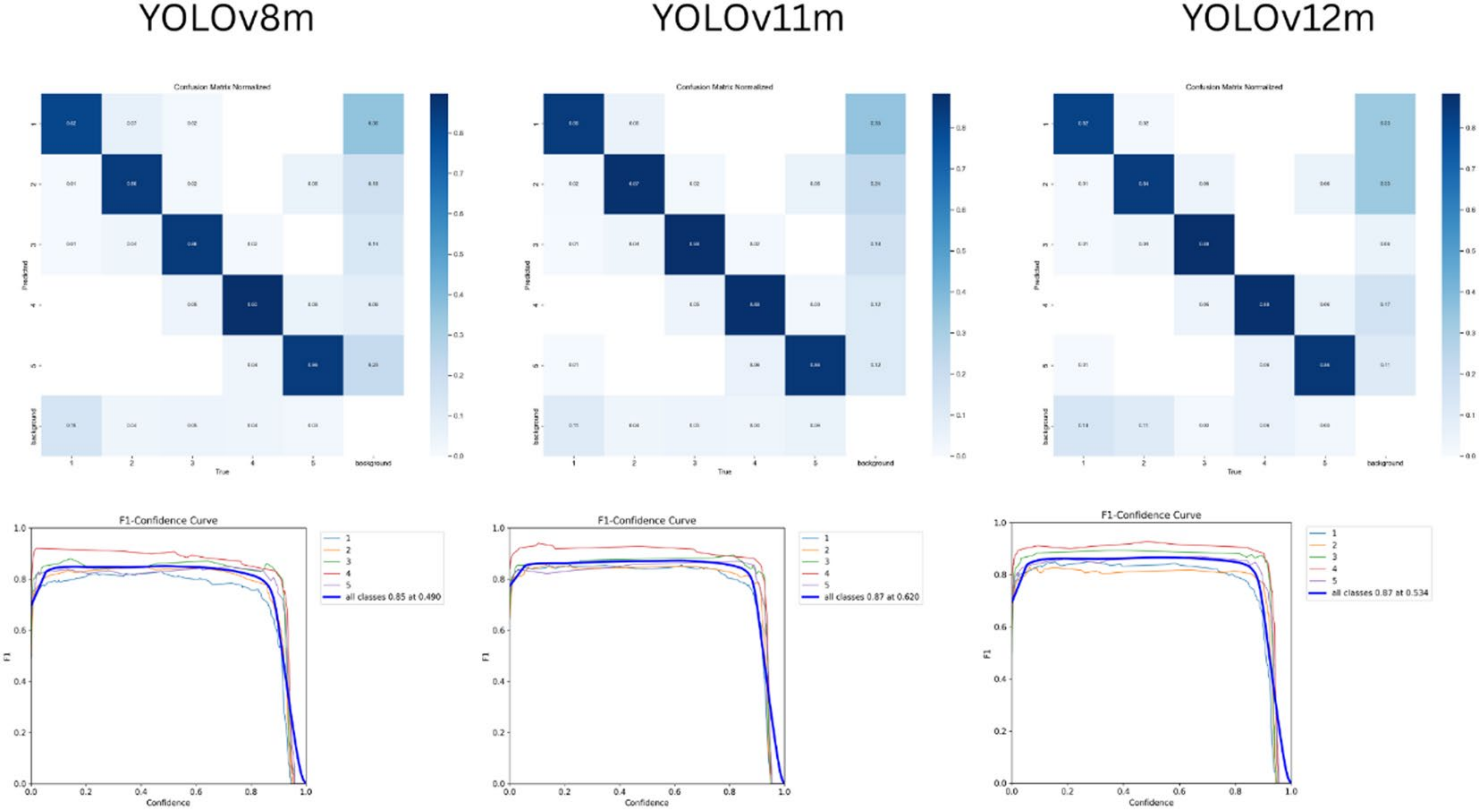
Normalized confusion matrix and per-class F1 scores for YOLOv8m/11m/12m on the testing dataset.

**Fig. 5:**
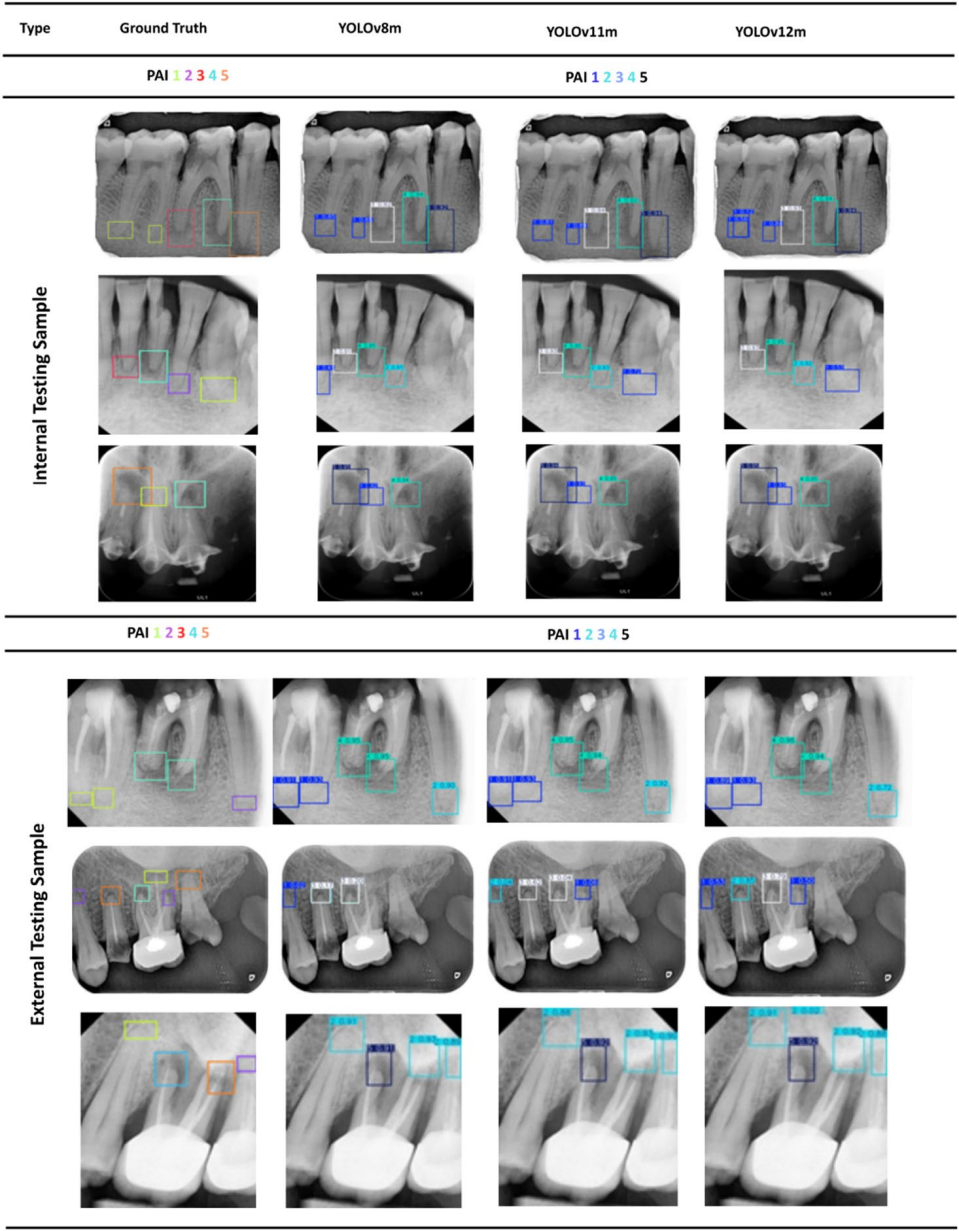
Qualitative comparison of PAI detection and classification for the internal testing set and the external testing samples. The ground truth is on the left, followed YOLOv8m, YOLOv11m, and YOLOV12m from left to right.

To aid comparison among models, Fig. 4 summarizes the confusion matrices and per-class F1 scores for YOLOv8m/11m/12m, allowing class-level distinctions to be visualized. Figure 5 provides qualitative overlays (model labels vs. ground truth) for representative cases across all PAI classes, illustrating areas of agreement and characteristic errors for both the testing and validation datasets.

Regarding performance, the tested models achieved comparably similar mAP50 of 86.4% for YOLOv8m and 86.6% for both YOLOv11m and YOLOv12m, with higher precision for YOLOv11m and YOLOv12m (88.5% and 89.1%, respectively) compared to YOLOv8m (86.8%). YOLOv11m had the highest recall (86.2%) and max F1 score (87.1%). Analysis of the confusion matrix on the testing dataset showed that all of the tested algorithms predicted classes 3–5 better than classes 1 and 2. An outperformance was noticed for YOLOv11m in predicting classes 1 and 2, and for YOLOv8m in predicting class 4. While for class 5 all algorithms had the same true positive rate.

The qualitative comparison of the prediction capabilities of all models on internal and external datasets is show in Fig. 5. The first column shows the ground truth followed by columns displaying the predictions from YOLOv8m/11m/12m. On the internal testing dataset, the models show qualitative agreement. Predicted boxes align tightly with ground-truth annotations and PAI scores with very few mistakes. Localization is precise around apices, duplicates are rare, and performance is consistent across YOLO variants.

On the external test set, performance remains usable and mostly reliable, however, there are signs of domain shift. High score lesions are correctly predicted, but there are under-detection of small/low-contrast lesions and grade confusions between adjacent PAI scores. Also, there are slight localization drift around multi-rooted teeth. These errors suggest the need for more diverse, multi-source training data, stronger geometry augmentations and hard-negative mining, plus class-aware thresholds to improve generalization.

The descriptive performance metrics were complimented with mean per-image F1 and 95% confidence intervals (CIs) obtained via image-level bootstrap (10,000 resamples; percentile method). This was performed on the full 85 test set images. To compare models on lesion-level outcomes, McNemar’s exact test (two-sided) was conducted because it evaluates paired binary outcomes on the same ground-truth lesions without distributional assumptions. For each lesion, “success” was defined as at least one detection of the correct class with IoU ≥ 0.5 after NMS and a fixed confidence threshold; subsequently followed by the computation of the a/b/c/d contingency and the exact binomial tail p-value.

The three models exhibit high and broadly consistent image-level F1 (Table 2), with substantial CI overlap, indicating only modest differences. YOLOv11m attains the highest point estimate (0.8596) and its CI is slightly shifted upward relative to YOLOv8m and YOLOv12m, suggesting a small practical advantage; however, CI overlap means this advantage may not be statistically significant at the image level.

**Table 2:** Mean F1 with 95% bootstrap CIs (internal test).

Model	Mean F1	95% CI
YOLOv8m	0.8307	0.7883–0.8696
YOLOv11m	0.8596	0.8194–0.8968
YOLOv12m	0.8537	0.8126–0.8912

The McNemar analysis is presented in Table 3. Across 534 ground-truth lesions, YOLOv11m outperformed YOLOv8m (*p* = 0.0015) and YOLOv12m (*p* = 0.0161), driven by many more YOLOv11m-only correct detections than the reverse (20 vs. 4, and 22 vs. 8, respectively). YOLOv8m and YOLOv12m were statistically indistinguishable (*p* = 0.864). Considering three pairwise tests, both YOLOv11m advantages remain significant under Holm–Bonferroni correction. Overall, image-level F1 suggests broadly comparable performance with YOLOv11m as the numerical front-runner, and lesion-level McNemar tests corroborate a significant edge for YOLOv11m over the other two models.

**Table 3:** Lesion-level paired comparisons (McNemar’s exact test, two-sided; success = correct class with IoU ≥ 0.5).

Pair	Success rate (A, B)	Discordant (A-only, B-only)	*p*-value
YOLOv8m vs. YOLOv11m	0.8352, 0.8652	4, 20	0.001544
YOLOv8m vs. YOLOv12m	0.8352, 0.8390	16, 18	0.864166
YOLOv11m vs. YOLOv12m	0.8652, 0.8390	22, 8	0.016125

### Post-hoc power analysis

To assess adequacy of the dataset size, we complemented bootstrap confidence intervals with a post-hoc power analysis. Specifically, we considered the observed mean F1-scores on the internal test set as the primary outcome and compared them against chance-level performance (F1 ≈ 0.20 for five PAI categories). Post-hoc power analysis indicated that all three models achieved greater than 99% power at α = 0.05 to detect performance above chance (F1 ≈ 0.20). The bootstrap 95% CIs (YOLOv8m: 0.7883–0.8696; YOLOv11m: 0.8194–0.8968; YOLOv12m: 0.8126–0.8912) exclude chance-level performance, further confirming that the dataset was sufficient to demonstrate meaningful classification capability.

## Discussion

Previous studies utilizing periapical radiographs adopted a binary approach, focusing solely on the presence or absence of radiolucency^33,34^. Only one study^24^ expanded on this by adding a classification task to further elaborate on the health/disease state of periapical tissues, using the PAI scoring system and an earlier version of YOLO algorithms. The current study sought to develop a DL model, performing the same tasks, using the latest YOLO algorithms and assess the impact of their novel architectural advancements on model performance in terms of accuracy and speed.

For DL models to be generalizable, they require a substantial amount of annotated data that should be as heterogeneous as possible^35,36^. This is a common limitation of earlier studies, where data were often sourced from a single center^18,22,23^. The question of what constitutes an “ideal” sample size for image datasets in DL applications remains open, with most studies lacking formal sample size calculations; indeed, no formal sample size calculation was performed in most of the studies. Therefore, active learning, self-supervised learning techniques, and creation of synthetic data were suggested to augment small datasets thereby reducing the time and labor needed for annotation^36,37^.

The present study’s approach to sample size and data collection demonstrates both strengths and limitations. While no formal sample size calculation was performed, the researchers employed alternative strategies to enhance the study’s robustness and generalizability. Heterogeneity was achieved by collecting data from different centers utilizing different digital imaging modalities; This was complemented by broad inclusion criteria of radiographs obtained before, during and after endodontic treatment for all teeth. Moreover, image augmentation techniques were applied to the training dataset during pre-processing to increase its size tenfold (10X) by applying random transformations to the annotated images^38^. This particular step is crucial as it balances the training dataset to prevent bias towards majority classes^15^ and forces the model to learn objects in new locations, and against different surrounding pixels. No attempt was made to increase the size of the initial dataset as pilot computations generated high accuracy metrics. Future model-fine tuning can incorporate additional data to enhance its generalizability. Furthermore, a post-hoc analysis of the model performance was conducted and supported the adequacy of the dataset size, thereby reinforcing the validity of the study’s conclusions despite the lack of a priori sample size determination.

Models were trained using the medium (m) variant of YOLOv8, YOLOv11, and YOLOv12 algorithms, considered state-of-the-art object detection and classification algorithms. A key innovation of YOLO is its ability to perform real-time object detection in a single pass through the neural network, making it remarkably fast and efficient. Unlike traditional CNNs like Mask R-CNN, which use complex multi-stage pipelines, YOLO uses a single unified model for both region proposal and classification^39^. The study used the medium variants of YOLOv8/11/12, which apply intermediate depth/width scaling within each family. Pilot ablations indicated no significant advantage of larger (l/x) models over (m) yet increased compute requirement; therefore, (m) provided the best accuracy-efficiency trade-off for our dataset and target runtime.

A key advantage of YOLOv8, over its predecessors is its anchor-free design, meaning the model predicts the center of an object rather than the offset from a comprehended anchor box. This approach reduces the number of box predictions, leading to faster data processing during training. Moreover, it incorporates several architectural improvements and redesigned optimizations to process input images more effectively. This included newer types of convolutional layers in the backbone that utilise advanced activation functions for faster computation and better generalisation, a more sophisticated version of the Featured Pyramid Network (FPN) in the head which integrates additional pathways and skip connections to enhance the information flow between the layers and better utilise the multi-scale features generated by the backbone, as well as the use of newer loss functions and anchor optimization techniques in the neck for effective bounding box regression and class prediction.

Building on this foundation, YOLOv11 and YOLOv12 have introduced advanced modules and attention mechanisms, significantly boosting detection and segmentation performance. YOLOv11 features the C3k2 block, SPPF for multi-scale features, and C2PSA for enhanced spatial attention^27^. YOLOv12 further advances the architecture by integrating Area Attention, R-ELAN for efficient aggregation, and Flash Attention for optimized memory access^28^.

A comparison of model architecture amongst the tested algorithms revealed that YOLOv11m and YOLOv12m had more layers. This allows for hierarchical feature learning in which the early layers capture simple features as straight lines and edges while deeper layers learn more complex patterns as composite shapes and objects. From the clinical standpoint, this suggests a capability to capture more image details. This was reflected in their higher max F1 score (87.1% and 86.6%, respectively) in comparison to YOLOv8m (85.2%). Regarding the number of trainable parameters, YOLOv11m and YOLOv12m had fewer parameters. This resulted in consumption of less GFLOPs and time during training which is a computational advantage, Also, fewer parameters often act as a form of regularization by limiting complexity and allowing for more iterations and experimentation with hyperparameters.

In the domain of object detection, all models performed similarly, with YOLOv11m achieving the highest Recall suggesting better identification of actual positive cases, whereas YOLOv12m achieved the highest Precision suggesting a reduced incidence of false positive predictions. Clinically, both metrics are crucial for accurate disease identification during the diagnostic process.

Analysis of the confusion matrices of the trained models revealed that YOLOv11m excelled in the detection and classification of scores 1 and 2, underscoring the impact of its architectural improvements on feature extraction and data representation. Conversely, YOLOv8m excelled in detecting class 4 suggesting effective learning or data representation of this class. YOLOv12m, however, showed mixed results with declines in class 2 detection and stability in the detection of other classes, suggesting that recent modifications in the model architecture may not have been beneficial for all classes. It is likely that recent modifications in YOLOv12 may trade sensitivity toward higher-contrast patterns, slightly reducing margin on borderline score 2 cases. In comparison, YOLOv11m’s more conservative, well-established detection head and PAN/FPN feature aggregation (decoupled cls/reg and a mature training recipe) appear better calibrated on our dataset size, yielding more uniform class-wise behavior.

Finally, PAI scores 4 and 5 had the highest true predictions and minimal misclassifications. This is attributed to the more advanced stages of periapical lesions, where the pathological changes in the alveolar bone are more pronounced and easily distinguishable from healthy tissue. The well-defined nature of these lesions and the significant difference in pixel density creates a clear contrast in radiographic images, contributing to the minimal misclassifications observed. In contrast, the models had a weaker performance for PAI scores 1 and 2. Although the distinction between them is theoretically desirable for early disease detection, yet former outcome studies^40,41^. dichotomized these scores as “healthy”. The semantic difference between scores 1, 2, and the background healthy alveolar bone may need clearer distinction in future studies.

The findings of the present study, despite the challenges in making direct comparisons, align with the broader literature. These challenges stem from variations in DL approaches, datasets, and performance metrics across different studies. Nevertheless, the results are consistent with a recent meta-analysis which reviewed 12 studies and reported sensitivities ranging from 0.65 to 0.96 for detecting radiographic periapical lesions using DL algorithms^42^. Moreover, the outcomes of the current study agree with earlier research^18,42^, even though those studies concentrated on panoramic and computed tomographic images rather than the digital periapical films used in this investigation^18,43^.

Limitations of the current study include the initial dataset size which may impact the model’s ability to generalize and pose a prospect of overfitting and reduced transferability. Small datasets may not capture the diversity of all clinical scenarios. This can be addressed by acquiring more images to either retrain the proposed models from scratch or use the current model weights to perform transfer learning.

Moreover, small datasets may have an under representation of some classes and an over representation of others. There was a potential imbalance in the distribution of PAI scores among tooth types and locations with an over representation of mandibular and maxillary molars in our initial dataset. Although this reflects their clinical prevalence amongst teeth requiring root canal treatment, our attention was directed to the periapical region of the teeth and their associated PAI score rather than the tooth type. Ideally, a balanced representation of all variables including tooth types in addition to PAI scores would avoid false predictions and misleading metrics. Though difficult to achieve, this should be pursued in future studies.

Another limitation of our model is that it was trained on radiographs acquired from permanent teeth with mature roots only. This would produce false positive predictions in cases of immature teeth with open apices and should be addressed in future research.

## Conclusion

This study highlights the significant potential of modern YOLO one-stage object detection algorithms in assessing periapical tissue conditions using the PAI scoring system. Through rigorous evaluation and comparison, these models have demonstrated robustness and accuracy, with YOLOv11m standing out for its balanced performance and processing efficiency. YOLOv11m effectively balances detection accuracy with inference time, making it a preferred choice for clinical implementation. While YOLOv12m offers higher precision at a slower speed, YOLOv8m achieves rapid inference despite its complexity.

To ensure generalizability and readiness for clinical deployment, our best performing model, YOLOv11m, would benefit from refinements. Future efforts should focus on refining YOLOv11m by expanding training datasets, securing external validation from diverse sources, and implementing continuous performance monitoring alongside active learning strategies. These steps are essential to ensure the model’s reliability, generalizability, and long-term efficacy. By automating and improving the detection of periapical lesions, these algorithms could greatly enhance the efficiency and accuracy of periapical lesion detection in dental practice, ultimately leading to better clinical outcomes. We also recommend that future research explore and assess LLM-based AI Agents and workflows that can be integrated in clinical settings.

## Supplementary Information

Below is the link to the electronic supplementary material.

Supplementary Material 1

## Data Availability

All data supporting the findings of this study are available within the paper and its Supplementary Information.
