# Supplementary material for: Automated assessment of periapical health based on the radiographic periapical index using YOLOv8, YOLOv11, and YOLOv12 one-stage object detection algorithms
Source: Sci Rep. 2025 Oct 20;15:36487. doi: 10.1038/s41598-025-21761-5 (PMC12537981; doi:10.1038/s41598-025-21761-5)
Supplement: Supplementary file 1 — Supplementary Material 1 [file 41598_2025_21761_MOESM1_ESM.docx]

**Supplementary Table 1:** Distribution of PAI scores in the training, validation, and testing datasets before augmentation according to tooth type (anterior, premolar, molar) in the maxilla and mandible.

|  | **Maxilla** | | | | | | | | | | | |
| --- | --- | --- | --- | --- | --- | --- | --- | --- | --- | --- | --- | --- |
|  | **Anterior** | | | | **Premolars** | | | | **Molars** | | | |
| **PAI** | **Training** | **Validation** | **Test** | **Total** | **Training** | **Validation** | **Test** | **Total** | **Training** | **Validation** | **Test** | **Total** |
| **1** | 50 | 14 | 10 | **74** | 64 | 25 | 7 | **96** | 95 | 42 | 22 | **159** |
| **2** | 30 | 6 | 3 | **39** | 42 | 9 | 7 | **58** | 55 | 16 | 7 | **78** |
| **3** | 18 | 9 | 3 | **30** | 18 | 10 | 3 | **31** | 43 | 11 | 5 | **59** |
| **4** | 12 | 6 | 4 | **22** | 12 | 4 | 3 | **19** | 15 | 8 | 6 | **29** |
| **5** | 16 | 6 | 8 | **30** | 15 | 2 | 2 | **19** | 17 | 10 | 6 | **33** |
| **Total** | **160** | **51** | **32** | **243** | **191** | **59** | **24** | **274** | **263** | **101** | **51** | **415** |
|  | **Mandible** | | | | | | | | | | | |
|  | **Anterior** | | | | **Premolars** | | | | **Molars** | | | |
| **PAI** | **Training** | **Validation** | **Test** | **Total** | **Training** | **Validation** | **Test** | **Total** | **Training** | **Validation** | **Test** | **Total** |
| **1** | 16 | 5 | 5 | **26** | 67 | 16 | 4 | **87** | 174 | 68 | 28 | **270** |
| **2** | 14 | 4 | 3 | **21** | 60 | 20 | 3 | **83** | 186 | 53 | 30 | **269** |
| **3** | 13 | 3 | 3 | **19** | 15 | 2 | 0 | **17** | 142 | 45 | 28 | **215** |
| **4** | 3 | 3 | 3 | **9** | 10 | 4 | 2 | **16** | 139 | 51 | 26 | **216** |
| **5** | 8 | 2 | 1 | **11** | 8 | 2 | 0 | **10** | 149 | 35 | 20 | **204** |
| **Total** | **70** | **20** | **17** | **107** | **189** | **50** | **15** | **254** | **989** | **322** | **160** | **1471** |
